# Supplementary material for: Transitions between phyllotactic lattice states in curved geometries
Source: Sci Rep. 2020 Oct 15;10:17411. doi: 10.1038/s41598-020-74158-x (PMC7566608; doi:10.1038/s41598-020-74158-x)
Supplement: Supplementary file 1 — Supplementary information 1. [file 41598_2020_74158_MOESM1_ESM.pdf]

# Supplementary Information

# Transitions between phyllotactic lattice states in curved geometries

**H. S. Ansell[1], A. A. Tomlinson[2], and N. K. Wilkin[2,*]**

[1]Department of Physics and Astronomy, University of Pennsylvania, Philadelphia, PA, 19104, USA
[2]School of Physics and Astronomy, University of Birmingham, Birmingham, B15 2TT, UK

*Correspondence to n.k.wilkin@bham.ac.uk

## A $\alpha$ values at transitions

Table S1 states the transition values $\alpha_T$ at which the lattice state transitions into the given phyllotactic state as the value of $\alpha$ is increased from $\alpha = 0$. The transition values are calculated for the case of cylindrical confinement. This table is a reproduction of results presented previously in Ref. [20] of the main text, extended here to $\alpha = 100.824$.

| $\alpha_T$ | State | $\alpha_T$ | State | $\alpha_T$ | State |
|---|---|---|---|---|---|
| 0 | (1, 1, 0) | 30 | (6, 3, 3) | 68.0683 | (9, 5, 4) |
| 2 | (2, 1, 1) | 31.7613 | (6, 4, 2) | 71.604 | (9, 6, 3) |
| 4 | (2, 2, 0) | 34.0042 | (6, 5, 1) | 73.3 | (8, 8, 0) |
| $8\sqrt{3/5}$ | (3, 2, 1) | 38.7034 | (6, 6, 0) | 75.7325 | (9, 7, 2) |
| 9.1912 | (3, 3, 0) | $504/\sqrt{143}$ | (7, 4, 3) | 80.7751 | (9, 8, 1) |
| 12 | (4, 2, 2) | 43.9001 | (7, 5, 2) | 85.4282 | (10, 5, 5) |
| 14.4506 | (4, 3, 1) | 47.2565 | (7, 6, 1) | 87.1823 | (10, 6, 4) |
| 16.7283 | (4, 4, 0) | $416/3\sqrt{7}$ | (8, 4, 4) | 89.5129 | (10, 7, 3) |
| $160/3\sqrt{7}$ | (5, 3, 2) | 56 | (7, 7, 0) | 92.3081 | (9, 9, 0) |
| 23.0956 | (5, 4, 1) | 58.4359 | (8, 6, 2) | 95.341 | (10, 8, 2) |
| 26.5641 | (5, 5, 0) | 62.8623 | (8, 7, 1) | 100.824 | (11, 6, 5) |

**Table S1.** Transition values $\alpha_T$ for which the system adopts the given phyllotactic state as the value of $\alpha$ is increased.

## B Comparison between molecular dynamics and Monte Carlo results

In this section we give a direct comparison between results obtained for ground states using molecular dynamics (MD) and Monte Carlo (MC) methods. We use as our example the transition between the $(4,3,1)$ and $(4,4,0)$ states, which is expected to occur at $\alpha = 16.73$. Figures S1(a) and S2(a) show snapshots of the ground states obtained using MD and MC respectively. In each snapshot there are 130 vortices and the system dimensions are $L = 50$, $c_0 = 6.0$ and $\Delta c = 0.5$.

In Figs. S1(b) and S2(b) we plot the density variation within each system, while in Figs. S1(c) and S2(c) we plot the resulting variation in the parameter $\alpha$. The dark grey dashed lines in each graph correspond to the position of the transition within the system, which is marked by the presence of a single dislocation in the system snapshots. The light grey lines on each plot represent the expected $\alpha$ value of the transition and the corresponding position within the system at which the local value of $\alpha$ takes on that value, which is calculated from the red best-fit curve. In both cases, the difference between the expected and actual positions of the transition within the system corresponds to less than 4% of the system length. This difference is the length of the average lattice parameter in the snapshots.

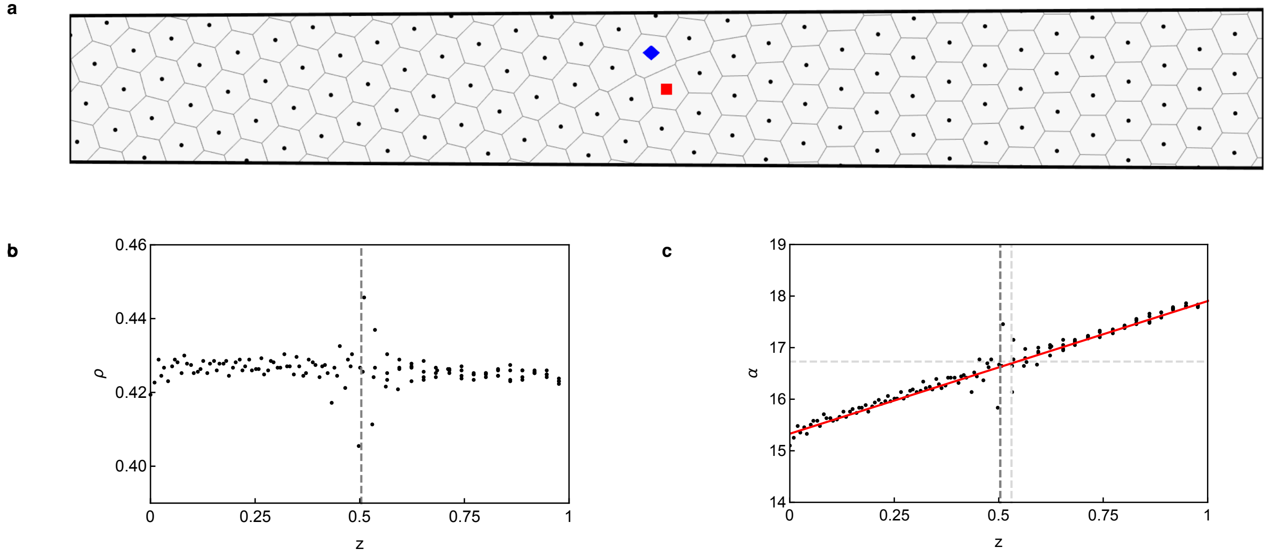


**Figure S1.** Ground state structure obtained using MD methods. (a) Snapshot of the system showing the $(4,3,1)$ to $(4,4,0)$ transition. (b) Local density variation throughout the system. The dashed grey line indicates the position of the transition. (c) Local $\alpha$ variation throughout the system. The dark grey line indicates the position of the transition while the lighter lines indicate the expected $\alpha$ value of the transition and its corresponding position within the system.

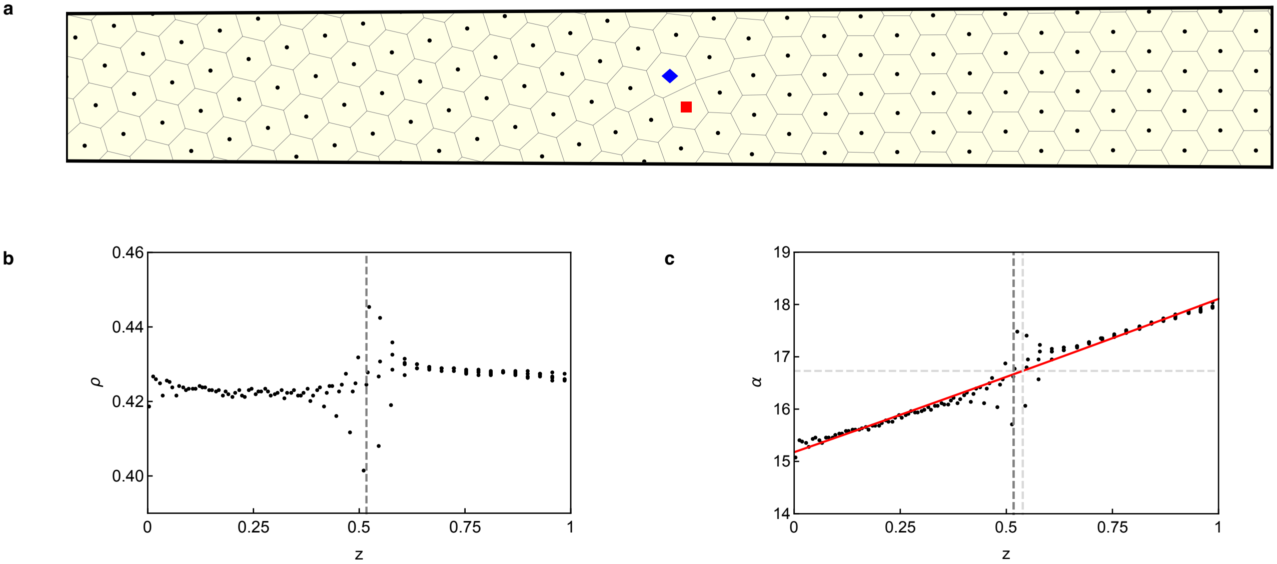


**Figure S2.** Ground state structure obtained using MC methods. (a) Snapshot of the system showing the $(4,3,1)$ to $(4,4,0)$ transition. (b) Local density variation throughout the system. The dashed grey line indicates the position of the transition. (c) Local $\alpha$ variation throughout the system. The dark grey line indicates the position of the transition while the lighter lines indicate the expected $\alpha$ value of the transition and its corresponding position within the system.

## C Molecular dynamics on the horn

We parameterise the horn surface using cylindrical coordinates $(r,\theta,z)$, with positions on the surface described by $\mathbf{r} = (r(z)\cos\theta, r(z)\sin\theta, z)$. The surface has a profile

$$r(z) = \frac{r_0}{\sqrt{1-\eta z}} \tag{C.1}$$

where $r_0 = c_0/(2\pi)$ is the radius at $z=0$ and $z$ is the fractional distance along the length of the surface, such that $0<z<1$, and $\eta > 0$.

Throughout this section subscripts $z$ or $\theta$ indicate a derivative with respect to that variable, while subscripts $i$ and $j$ are used to index particular vortices. It is useful to note the following relations

$$r_z(z) = \frac{r_0\eta}{2(1-\eta z)^{3/2}} = \frac{\eta}{2r_0^2} r(z)^3 \tag{C.2}$$

$$r_{zz}(z) = \frac{3r_0\eta^2}{4(1-\eta z)^{5/2}} = \frac{3\eta^2}{4r_0^4} r(z)^5. \tag{C.3}$$

In order to do molecular dynamics, we need to know the geodesics on the surface between pairs of points. The distance $d_{ij}$ between a pair of vortices is the length of the geodesic connecting the pair along the surface, a derivation for which is given in the following section. We numerically solve for the geodesics and calculate $d_{ij}$ for each pair within the cut-off radius.

The net force on a chosen vortex in a given time step is the sum of the individual forces acting upon it. We set a distance length scale $a_0$ as the length of the lattice parameter for the expected state at $z=0.5$, i.e. $f_{ij} = -f_0 K_1(d_{ij}/a_0)$, where $f_{ij}$ is the magnitude of the force on vortex $i$ due to vortex $j$, $f_0 = 1$ is a constant and $K_1$ is a modified Bessel function of the second kind. The distance $\delta d$ moved by a vortex is restricted to a small value through the choice of the value of time step $\delta t$. We therefore approximate that the vortex moves a distance $\delta d$ along the tangent vector to the net force, which is approximately parallel to the geodesic along the direction of the net force over the distance $\delta d$.

### C.1 Determining the distance between two particles - boundary value problem

The distance between two points is determined by numerically calculating the length of the geodesic connecting the pair. Depending on the state on the surface, there may be occasions where either $\theta_z$ or $z_\theta$ is singular. As such, geodesic equations for both $\theta(z)$ and $z(\theta)$ must be known so that if one has a singularity, the other can be used.

#### *C.1.1 Solving for* $\theta(z)$

We determine the equation satisfied by the geodesic $\theta(z)$ on the surface. The line element $\mathrm{d}s$ is given by

$$\mathrm{d}s = \sqrt{\mathrm{d}r^2 + r^2\,\mathrm{d}\theta^2 + \mathrm{d}z^2} \tag{C.4}$$

$$= \mathrm{d}z\sqrt{r_z^2 + r^2\theta_z^2 + 1} \tag{C.5}$$

$$\equiv \mathrm{d}z\, f \tag{C.6}$$

where we have dropped the functional dependence $r = r(z)$ for brevity.

The geodesic must satisfy the Euler-Lagrange equation for $\theta$, leading to:

$$0 = \frac{\mathrm{d}}{\mathrm{d}z}\frac{\partial f}{\partial \theta_z} \tag{C.7}$$

$$= \frac{2rr_z\theta_z + r^2\theta_{zz}}{\sqrt{1+r_z^2+r^2\theta_z^2}} - \frac{r^2\theta_z(r_zr_{zz} + rr_z\theta_z^2 + r^2\theta_z\theta_{zz})}{(\sqrt{1+r_z^2+r^2\theta_z^2})^3} \tag{C.8}$$

Using that $\sqrt{1+r_z^2+r^2\theta_z^2} > 0$, we can multiply out the denominator to give

$$0 = (2rr_z\theta_z + r^2\theta_{zz})(1+r_z^2+r^2\theta_z^2) - r^2\theta_z(r_zr_{zz} + rr_z\theta_z^2 + r^2\theta_z\theta_{zz}) \tag{C.9}$$

$$0 = r\left[r(1+r_z^2)\theta_{zz} + r^2r_z\theta_z^3 + (2r_z(1+r_z^2) - rr_zr_{zz})\theta_z\right] \tag{C.10}$$

We make use of Eqs. (C.1)-(C.3) leading to

$$0 = \left(1 + \frac{\eta^2r^6}{4r_0^4}\right)\theta_{zz} + \frac{\eta r^4}{2r_0^2}\theta_z^3 + r^2\left(\frac{\eta}{r_0^2} - \frac{\eta^3r^6}{8r_0^6}\right)\theta_z \tag{C.11}$$

Substituting in for $r(z)$ then leads to the equation satisfied by the geodesic $\theta(z)$

$$0 = 2(1-\eta z)(4(1-\eta z)^3 + \eta^2 r_0^2)\theta_{zz} + 4\eta r_0^2(1-\eta z)^2\theta_z^3 + \eta(8(1-\eta z)^3 - \eta^2 r_0^2)\theta_z \tag{C.12}$$

Equation (C.12) can be solved numerically, with boundary conditions given by the positions of the two vortices, to find $\theta(z)$ and $\theta_z(z)$. The distance $d_{ij}$ between vortices $i$ and $j$ is then the length of the geodesic and is determined by numerically integrating

$$d_{ij} = \int \mathrm{d}s = \int_{z_i}^{z_j} \mathrm{d}z \sqrt{1 + r_z(z)^2 + r(z)^2\theta_z(z)^2}. \tag{C.13}$$

### C.1.2 Solving for $z(\theta)$

We repeat the process from the last section, this time solving for the geodesic $z(\theta)$. In this case the line element d$s$ is given by

$$\mathrm{d}s = \mathrm{d}\theta \sqrt{r(z(\theta))^2 + z_\theta^2 + r_\theta(z(\theta))^2} \tag{C.14}$$

$$= \mathrm{d}\theta \sqrt{\frac{r_0^2}{1-\eta z} + z_\theta^2 + \frac{r_0^2\eta^2 z_\theta^2}{4(1-\eta z)^3}} \tag{C.15}$$

$$\equiv \mathrm{d}\theta\, g \tag{C.16}$$

Solving the Euler-Lagrange equation for $z$ gives

$$\frac{\partial g}{\partial z} = \frac{\mathrm{d}}{\mathrm{d}\theta}\frac{\partial g}{\partial z_\theta} \tag{C.17}$$

$$\frac{\partial g}{\partial z} = \frac{\frac{3\eta^3 r_0^2 z_\theta^2}{4(1-\eta z)^4} + \frac{\eta r_0^2}{(1-\eta z)^2}}{2\sqrt{\frac{\eta^2 r_0^2 z_\theta^2}{4(1-\eta z)^3} + \frac{r_0^2}{1-\eta z} + z_\theta^2}} \tag{C.18}$$

$$\frac{\mathrm{d}}{\mathrm{d}\theta}\frac{\partial g}{\partial z_\theta} = \frac{\frac{\eta^2 r_0^2 z_{\theta\theta}}{2(1-\eta z)^3} + \frac{3\eta^3 r_0^2 z_\theta^2}{2(1-\eta z)^4} + 2z_{\theta\theta}}{2\sqrt{\frac{\eta^2 r_0^2 z_\theta^2}{4(1-\eta z)^3} + \frac{r_0^2}{1-\eta z} + z_\theta^2}} - \frac{1}{4}\left(\frac{\eta^2 r_0^2 z_\theta}{2(1-\eta z)^3} + 2z_\theta\right)\times$$
$$\left(\frac{3\eta^3 r_0^2 z_\theta^3}{4(1-\eta z)^4} + \frac{\eta r_0^2 z_\theta}{(1-\eta z)^2} + \frac{\eta^2 r_0^2 z_\theta z_{\theta\theta}}{2(1-\eta z)^3} + 2z_\theta z_{\theta\theta}\right)\left(\frac{\eta^2 r_0^2 z_\theta^2}{4(1-\eta z)^3} + \frac{r_0^2}{1-\eta z} + z_\theta^2\right)^{-3/2} \tag{C.19}$$

Simplifying this leads to the equation for the geodesic for $z(\theta)$:

$$0 = 2(1-\eta z)\left(4(1-\eta z)^3 + \eta^2 r_0^2\right)z_{\theta\theta} + \eta\left(\eta^2 r_0^2 - 8(1-\eta z)^3\right)z_\theta^2 - 4\eta r_0^2(1-\eta z)^2 \tag{C.20}$$

Equation (C.20) can be solved numerically to find $z(\theta)$ and $z_\theta(\theta)$. The distance $d_{ij}$ between a vortex pair is once again the length of the geodesic and is determined by numerically integrating

$$d_{ij} = \int \mathrm{d}s = \int_{\theta_i}^{\theta_j} \mathrm{d}\theta \sqrt{r(z(\theta))^2 + z_\theta(\theta)^2 + r_\theta(z(\theta))^2} \tag{C.21}$$

## D Determining the expected vortex locations on the horn

We derive the equation of the set of curves which can be used to construct the phyllotactic state with constant $\alpha$ on the curved surfaces. The vertices at which these curves intersect define the expected locations of vortex sites. The curves, known as *loxodromes*, always have their tangent vector at a fixed angle relative to the parallels and meridians of the surface. For the surface of revolution of the curve $r(z)$ about the $z$ axis, parameterised in cylindrical coordinates as $\mathbf{r} = (r(z)\cos\theta, r(z)\sin\theta, z)$, a set of orthonormal unit vectors along the surface can be defined as

$$\hat{\mathbf{e}}_\theta = \begin{pmatrix} -\sin\theta \\ \cos\theta \\ 0 \end{pmatrix} \qquad \hat{\mathbf{e}}_v = \frac{1}{\sqrt{1+r_z^2}}\begin{pmatrix} r_z\cos\theta \\ r_z\sin\theta \\ 1 \end{pmatrix} \tag{D.1}$$

where, in this section, subscript $z$ denotes a derivative with respect to $z$. The parallels of the surface are the lines of constant $v$ (or equivalently $z$) and the meridians are the lines of constant $\theta$.

A curve $\gamma(z)$ for which the tangent vector is always at some fixed angle $\beta$ relative to the meridians must always be parallel to the curve

$$\hat{\beta} = \cos\beta\,\hat{\mathbf{e}}_v + \sin\beta\,\hat{\mathbf{e}}_\theta. \tag{D.2}$$

The values of $\beta$ used to construct a state are determined by the chosen value of $\alpha$ and the corresponding lattice vector directions.

Solving $\hat{\mathbf{T}} = \hat{\beta}$, where $\hat{\mathbf{T}}$ is the unit tangent vector to $\gamma(z)$ means that $\gamma(z)$ must satisfy

$$\frac{1}{\sqrt{1+r_z^2+r^2\gamma_z^2}}\begin{pmatrix} r_z\cos\gamma - r\gamma_z\sin\gamma \\ r_z\sin\gamma + r\gamma_z\cos\gamma \\ 1 \end{pmatrix} = \frac{\cos\beta}{\sqrt{1+r_z^2}}\begin{pmatrix} r_z\cos\gamma \\ r_z\sin\gamma \\ 1 \end{pmatrix} + \sin\beta\begin{pmatrix} -\sin\gamma \\ \cos\gamma \\ 0 \end{pmatrix} \tag{D.3}$$

where $r(z)$ and $r_z(z)$ are defined in Eqs. (C.1) and (C.2) respectively.

Solving for $\gamma_z(z)$ leads to

$$\gamma_z(z) = \pm\tan\beta\,\frac{\sqrt{1+r_z(z)^2}}{r(z)}. \tag{D.4}$$

This expression is true for the loxodromes on any surface of revolution with a shape profile $r(z)$. We integrate this expression on the horn with the boundary condition that $\gamma(z=0)=0$ to give the solution

$$\gamma(z) = \frac{1}{3}\tan\beta\left[\sqrt{1+\frac{4}{\eta^2 r_0^2}} - \sqrt{1+\frac{4(1-\eta z)^3}{\eta^2 r_0^2}} - \tanh^{-1}\left(\sqrt{1+\frac{4}{\eta^2 r_0^2}}\right) + \tanh^{-1}\left(\sqrt{1+\frac{4(1-\eta z)^3}{\eta^2 r_0^2}}\right)\right]. \tag{D.5}$$
